# Adopting open access in the social sciences and humanities: evidence from a developing nation

**DOI:** 10.1016/j.heliyon.2020.e04522

**Published:** 2020-07-23

**Authors:** Thu-Trang Vuong, Manh-Toan Ho, Minh-Hoang Nguyen, Thanh-Huyen T. Nguyen, Thanh-Dung Nguyen, Thi-Linh Nguyen, Anh-Phuong Luong, Quan-Hoang Vuong

**Affiliations:** a Sciences Po Paris, 75337 Paris, France; b Centre for Interdisciplinary Social Research, Phenikaa University, Yen Nghia Ward, Ha Dong District, Hanoi, 100803, Viet Nam; c A.I. for Social Data Lab, Vuong & Associates, 3/161 Thinh Quang, Dong Da District, Hanoi, 100000, Viet Nam; d Foreign Trade University, 91 Chua Lang Street, Dong Da District, Hanoi 100000, Viet Nam

**Keywords:** Social science, Information science, Open access, Scientific publishing, Social sciences and humanities, Vietnam

## Abstract

Open Access (OA) publishing, with ambitious movements such as Plan S, is engendering radical changes among academic publishers. Emerging countries need to keep publishing as well as adopt open access to catch up with the changes. Using exclusive data from the Social Sciences & Humanities Peer Awards (SSHPA) database, the study employed both descriptive statistics and a Bayesian linear regression model to examine the journals and publishers in which Vietnamese social scientists published during the period 2008–2019, and the potential of pursuing the OA movement in Vietnam. We found an increasing diversification in the publishing sources of Vietnamese social science researchers with growth rates of 9.8% and 14.1% per annum in the number of publishers and journals, respectively. Given that the proportion of Gold OA articles had a fourfold increase over the examined period, it seems that the Vietnamese academic community is adopting OA. Furthermore, Bayesian analysis results hint at positive associations of internal and external collaborative power (number of domestic and foreign authors, respectively) with the decision to publish in OA (*β*_b_TotalVN_OpenAccess_ = 0.22; *β*_b_TotalForeign_OpenAccess_ = 0.15). The results and its implications suggest that Vietnamese policymakers and university director boards should facilitate as well as control the quality of the scientific publishing and the OA movement.

## 1 Introduction

Since the establishment of the arXiv repository, the Open Access (OA) Movement has steadily gained attention and support from the scholarly community as well as from society at large. The launch of Plan S initiative in September 2018, which is promoted by cOAlition S, marked a milestone in achieving OA science (cOALition, 2020). It sets an ambitious goal that all scholarly publications funded by its members must be published open access (cOALition, 2020; Else, 2019; Noorden, 2020; Rabesandratana, 2019; Vuong, 2020a). So far, the radical plan has been endorsed by major scientific funders in the world. In the beginning, national funding agencies in Europe fully supported Plan S. Later on, international funders from other countries, Bill & Melinda Gates Foundation, Wellcome Trust joined with European funders (Brainard, 2020; cOALition, 2020). Currently, funders endorsing Plan S have contributed up to 6.4% of publications indexed in Web of Science (Quaderi et al., 2019).

Nonetheless, this goal is relatively ambitious for researchers and publishers to adopt (Else, 2019). As a result, the plan has been delayed by 2021, which hints at not only the tremendous challenge in pursuing OA publishing but also a great opportunity for scholarly communities in emerging countries. In the transition from mainly local scientific publishing to international publishing, being able to adopt the OA publishing framework would help emerging countries to catch up with the current global publishing standard.

Besides adopting the OA publishing framework, proactive government initiatives would be required to create a “publish or perish” culture in emerging countries, for the sake of meeting global publishing standards (Vuong, 2019a). In the “publish or perish” culture, publication count is undeniably one of the primary measures of a researcher's performance, and researchers are under pressure to publish to acquire a job, gain promotion and maintain their positions (Moosa, 2018). However, the pressure to publish has pushed early career researchers (ECRs), especially those from emerging countries, into becoming victims of “predatory journals” that exploit the OA framework but lack peer-review process and exist barely for profit rather than for science (Demir, 2018; Kurt, 2018). Publishing in such journals might result in not only low-quality control but also a loss of reputation and hard work of researchers. Therefore, understanding where scientists publish would offer emerging countries essential insights for future scientific research policies and the pursuit of Plan S's core ideas.

## 2 Literature review

Compared to decades ago, when emerging countries had a relatively high rate of studies published in domestic journals to those published in internationally indexed journals, academia has become globalized along with the world economy (Crew, 2019; Gaillard, 1992). Vietnam, with up to 77% of scientific output involved in international collaboration, can be considered as a typical contributor to the dramatic shift to scientific internationalization among emerging countries (Manh, 2015). This tremendous amount of international collaborated studies have raised the annual growth rate of scientific output to 17% (Nguyen et al., 2017).

Within this conjecture, after a long period of low scientific productivity and output, the fields of Social Sciences and Humanities (SS&H) have also witnessed phenomenal growth, largely thanks to the financial support from governmental and private organizations as well as policies promoting international collaboration and adoption of international publishing standard (Vuong et al., 2018b; Vuong, 2019b, 2020a).

Previously, there was no requirement for international publications from Vietnamese authorities. Nevertheless, the introduction of Circular 37/2014/TT-BKHCN in 2014, which requires all national projects to result in ISI/Scopus publications, and Circular 08/2017/TT-BGDĐT in 2017, which requires doctoral candidates to publish at least two articles in ISI/Scopus journals, has imposed enormous pressure on international publishing (Vietnam MOET, 2017; Nafosted, 2018; Nguyen et al., 2019; Vuong et al., 2020). As a result, these policies have been empirically linked with an increase in SS&H research productivity (Nguyen et al., 2019; Vuong, 2019b, 2020a).

Nevertheless, to be able to achieve global publishing standards, apart from an impressive scientific performance, the scientific quality and OA tendency among the Vietnamese scholarly community also need to be evaluated through journals in which articles are published. Currently, most studies focus on articles published in international journals rather than domestic ones, due to the low standards of Vietnamese SS&H journals. In comparison with Asian countries, Vietnamese journals lack scientific content, professional peer review, and publishing integrity (Tam, 2017). By 2015, Vietnam had 334 journals with authorized ISSN; however, only three of them, all of which belong to the field of natural sciences, are indexed in Scopus and none in ISI (S.H., 2016; Tam, 2017). There is no SS&H journal indexed in Scopus or ISI. It should be noted that two SS&H journals, *Journal of Asian Business and Economics Studies,* and *Journal of Economics and Development* are currently published by Emerald, tracking to become ISI/Scopus-indexed journals (Vuong, 2019b).

There have been few papers investigating the overall picture of scientific research in Vietnam regarding international collaboration and research output (Manh, 2015; Nguyen et al., 2017; Nguyen and Pham, 2011). Vietnam was among countries of low research output, with weaknesses such as heavy reliance on foreign co-authorship and limited research capacity at higher education institutions (Harman and Ngoc, 2010; Hien, 2010). Manh (2015) investigated Vietnam's scientific publications 1996–2013 using the Scopus database. Total output increased by 20% annually between 2002 and 2013. However, 77% of the output was from international collaboration. The list of top 20 journals in collaboration research reflected the dominance of medicine, biological and agricultural science, while the list of top 20 journals by domestic authors reflected the dominance of mathematics. Similarly, using the Web of Science database 2001–2015 of 18044 papers, Nguyen et al. (2017) found the rate of growth in scientific output annually 17%, ¾ of which was attributed to international collaboration. Nguyen and Pham (2011) found a strong relationship between scientific output and the extent of a knowledge economy, urging more investment from government and academic institutions by examining 165,020 articles in ISI-indexed journals of 10 Southeast Asia countries.

Overall, these papers are few and far between, lack updated data as well as in-depth analysis about the performance of Vietnamese researchers, particularly those in SS&H fields. As a result, the SSHPA database was built in 2017 to address these problems. The database is a comprehensive system from which accurate, updated, and focused information about the demographic characteristics and productivity of Vietnamese researchers with international publications in SSH fields can be produced (Vuong et al., 2018a).

None of the publications specifically focus on the quality of Vietnamese SS&H studies. Manh (2015) found that the quality of most popular journals based on international collaboration was higher than those of domestic authors, but the list is dominated by research in natural sciences. Only a few of them discuss the potential impact of the OA movement. Vuong (2019b) suggests the adoption of open science as a way to promote transparency. Currently, there is only one SS&H journal in the Directory of Open Access Journals (DOAJ) that belongs to the Vietnamese publisher, which is Da Lat University Journal of Science. However, this journal is not included in the SSHPA database because it is not in the official list of prestigious SS&H journals recognized by the Vietnam National Foundation for Science & Technology Development (Nafosted, 2018).

Collaboration in academic publishing has become the norm. Through collaboration, researchers can gain benefits from the increase in knowledge sharing, task specialization, work productivity, and visibility of research (Lee and Bozeman, 2005; Franceschet and Costantini, 2010; Vuong et al., 2017, 2018b). Previous studies in multiple disciplines found that the increasing number of co-authors led to a higher quality of scientific articles as measured by common proxies – journal's impact factor and the number of citations (Franceschet and Costantini, 2010; Larivière et al., 2014; Victor et al., 2016, pp. 1989–2013). The impact differs by types of collaboration and disciplines, with internationally co-authored articles generally getting more citations than domestically co-authored ones, and natural sciences gain more than social sciences (Bote et al., 2012; Frenken et al., 2010; Sooryamoorthy, 2009; Puuska et al., 2013).

Given the increasing international collaboration trend among developing countries, some studies have been conducted to examine this impact. International collaboration can lead to research with higher quality and visibility (Frenken et al., 2010). Studies have found that the majority of scientific output in Vietnam is attributed to international collaboration (Manh, 2015; Nguyen et al., 2017). Besides, the citation rate for internationally co-authored articles is higher than domestic articles (Confraria et al., 2017; Hien, 2010; Nguyen et al., 2017). However, what is lacking here is a distinction between types of collaboration in the field of social sciences.

The open-access model gives the audience free and unrestricted access to digital content of scholarly literature, which includes both peer-reviewed journals and unreviewed preprints (Gadd and Troll, 2016; Tenopir et al., 2016). There are currently many types of OA. In Gold OA, articles are published in an OA journal, and sometimes authors need to pay publication fees in the form of article processing charges or APC (Tenopir et al., 2016). In Green OA, articles are published in a toll-access journal but require self-archiving in an OA archive. Meanwhile, for hybrid OA, a subscription-based journal allows an article to be published open access with payment APC (Piwowar et al., 2018).

OA publishing serves to ensure equal access to knowledge and allows researchers around the world to contribute to scientific knowledge with considerably fewer financial barriers (Shuva and Taisir, 2016). On average, OA articles receive more downloads and citations compared with non-OA counterparts, four times and 1.6 times, respectively (Springer Nature, 2020). OA articles receive 18% more citations than average, largely attributed to the impact of Green and Hybrid OA.

Meanwhile, for the gold model, OA journals have the strong points of free access, visibility, and speed, but raise concerns about author charges, copyright, a perceived lack of prestige compared to traditional journals and the rise of OA predatory journals (Anderson, 2004; Coonin and Younce, 2010; Shuva and Taisir, 2016; Tenopir et al., 2016; Warlick and Vaughan, 2007). According to Tenopir et al. (2016), in choosing where to adopt OA publishing, the authors have to weigh between wide accessibility and the willingness to pay for APC charges.

In the pursuit of the Open Access movement in emerging countries, paying for APC is costly, which can hinder the adoption of OA publishing. According to (Shuva and Taisir, 2016), faculty members in developing countries only have modest salaries, so they cannot afford high APC charges. As a result, there might be a possibility that a higher number of co-authors per article might result in a higher likelihood of publishing in an Open Access journal. According to Luukkonen et al. (1992), researchers from less developed countries might regard international collaboration as a means of cost-sharing. Similarly, Manh (2015) stated that the importance of foreign collaboration was in part due to the limited research budget in Vietnam. Publishers also offer specific policies or programs to help authors to pay the APC. For instance, Elsevier has the Research4Life program (https://www.research4life.org), which cut down the publishing cost for authors from certain countries. Similarly, PLOS—one of the biggest Open Access publishers—also offers a similar program named PLOS Global Participation Initiative (https://plos.org/publish/fees/). Furthermore, Open Access publishers, such as MDPI, also provide Institutional Open Access Program (IOAP), which discounts the APC for researchers from partner institutions.

For that reason, we propose new models examining the determinants of domestic and international collaborative network expansions on scientific impact, measured by JIF, and the decision to publish in Open Access journal. The size of such collaborative networks is assumed to be a force, both internal and external, to foster improvement in output quality as well as the Open Access movement.

Accordingly, to assess the quality of studies and OA publishing trend as well as examine the impact of internal and external collaborative power on scientific quality and OA decision in Vietnam SS&H, our paper aims to investigate the following research questions:

1. What is the OA publication patterns of Vietnamese social scientists during the 2008–2019 period?
2. Do internal and external collaborative power (measured by the number of domestic and foreign authors, respectively) affect the decision to submit to OA journals?

## 3 Materials and methods

### 3.1 Materials

To examine where Vietnamese SS&H scholars publish their work, we use the Social Sciences & Humanities Peer Awards (SSHPA) database (URL: https://sshpa.com/). The database is a part of a national project, which aims to create a semi-automatic system to record the scientific output of Vietnamese researchers in the field of Social Sciences and Humanities since 2008. Since each data point corresponds to a publication and includes information regarding its journal and publishers, we are able to extract the information. The database's logical structure, the data collection process, and the data validation process were peer-reviewed and published in the article by Vuong (2018).

In terms of quality control, the SSHPA covers all articles published in international and national journals in the field of SS&H – which includes journals indexed in ISI Web of Science Core Collection, Scopus, or journals published by reputable publishers based on the official list as devised by the Vietnam National Foundation for Science & Technology Development (NAFOSTED) (Nafosted, 2018). Moreover, the SSHPA database collects the data daily, making it the most updated database regarding the scientific production of SS&H scientists in Vietnam. The data collection process also goes through several layers of quality control. Firstly, people who enter the data will list the attributes of the article. Then, the system will automatically find duplicates and other possible errors. Finally, an administrator will review the data for acceptance.

A comprehensive dataset of Vietnam SSH in the 2008–2019 period was extracted from the database. The final dataset is available in OSF (URL: https://osf.io/4mwqr/). Specifically, the following information was extracted from the dataset:

• Publisher, which refers to the agency that is listed as the publisher of journals, books, edited books, or conference proceedings, including commercial publishers, university publishers, society publishers, or even a conference organizer who publishes conference proceedings.
• Source, which refers to journals, books, edited books, or conference proceedings.
• Articles refer to journal articles, book chapters, or conference papers.

Furthermore, the number of Vietnamese authors, the number of foreign authors, and decision to submit to an Open Acess journal (see Table 1) were also employed for analysis:

**Table 1:** List of variables for Bayesian analysis.

Variable Type	Variable Names	Data Type	Description
Independent Variable	*TotalVN*	Ordinal Data	The number of Vietnamese authors in an article
	*TotalForeign*	Ordinal Data	The number of foreign authors in an article
Dependent Variable	*OpenAccess∗*	Binomial data (1 – Yes vs. 0 – No)	Whether an article is in an Open Access journal or not

We used the Unpaywall's Simple Query Tool (Accessible here: https://unpaywall.org/products/simple-query-tool) and Chrome's browser extensions to verify whether an article is open-access or not. Moreover, as Unpaywall covers articles with DOIs only, we manually searched for articles without DOIs on journals' websites and official repositories to identify their state of publications. Through answering these questions, we can assign the OA status accordingly:

• Is the article closed or open?
• Where is it hosted?
• Is it published in a fully OA source?
• Is it published under an open license?

In the dataset, the OA status was categorized into five types, which based on the categorization by Piwowar et al. (2018):

• Gold Access: an article that is published in a fully OA journal.
• Green Access: an article that is not accessible on its journal homepage but available in an official repository.
• Hybrid Access: an article that is published under an open license in a subscription journal.
• Bronze Access: a *gratis* OA article that is published in a subscription journal, but not under an open license for redistribution or reuse.
• Closed Access: an article that is published in a subscription journal, and inaccessible without subscription or fee.

It should be noted that for Bayesian analysis, we grouped Gold, Green, Hybrid, and Bronze types as Yes and Closed Access articles as No for the “*OpenAccess*” variable (Table 1).

### 3.2 Methods

In this paper, the authors employ both descriptive and Bayesian statistics to answer the research questions. The descriptive statistics are used to examine the following information extracted from the SSHPA database:

- Top publishers by the number of publications;
- The number of publishers and sources annually
- The number of new publishers and new sources annually;
- Top journals by the number of publications;
- The number of sources by their publishing models;

As these OA results are manually integrated into our original dataset, concerns over human errors are raised. To overcome this an ensure the dataset quality, we implement a cross-checking step in which data collectors will double-check the results collected by each other.

The Bayesian estimation is employed in the current study due to its advantages. First, the analysis follows Bayes' theorem that has no assumption of an infinite posterior data; the posterior distributions of parameters are simulated based on the prior distributions. They can be updated by conditioning on newly-observed data. Given the mounting criticisms on *p*-value's frailties (Halsey, 2019; Amrhein et al., 2019), the ability to present and visualize posterior distributions of Bayesian analysis provides more information for interpretation that facilitates reader's intuition and interpretation (McElreath, 2020). The *bayesvl* package, capitalizing on the current trends in Bayesian inference, is selected for statistical analysis (Vuong et al., 2020).

## 4 Results

### 4.1 Descriptive statistics

In general, there was a significant expansion of publishing outlets among Vietnamese social scientists during the 2008–2019 period. The database identified 19 books, 141 edited books, 21 conference proceedings, and 1188 journals that published 3122 publications. Not only did the number of publishers and journals grow dramatically.

#### 4.1.1 Publishers and journals

Among 1188 journals collected, 973 of them were published by ten well-known publishers (see Table 2). The top three publishers in terms of the number of publications, which were Elsevier, Taylor & Francis, and Springer, accounted for almost 50% of identified journals. Even though compared to Taylor & Francis, Elsevier had fewer journals in which Vietnamese social scientists were published, and it published the highest number of articles by Vietnamese scholars. As a young OA publisher, although MDPI barely provided publishing services to Vietnamese social scientists in 19 journals, it has successfully attracted 297 Vietnamese authors and 167 articles, ranking it sixth on the list.

**Table 2:** Top 10 publishers with the highest number of publications.

Publisher	Publications	Author	Journals
Elsevier	526	456	185
Taylor & Francis	500	460	207
Springer	422	523	180
Wiley	216	207	119
Emerald	195	195	94
MDPI	167	297	19
SAGE	122	164	71
Routledge	92	84	52
Cambridge University Press	52	48	33
BioMed Central (BMC)	48	89	13

**Figure 1:**
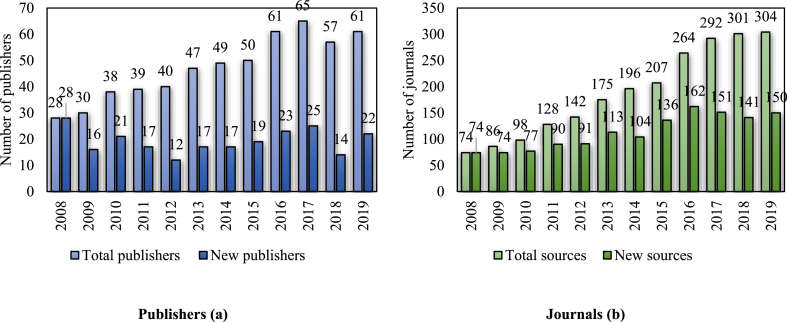
a) Total and new publishers from 2008 to 2019; b) Total and new sources from 2008 to 2019 (for all types of publication).

In choosing where to submit, Vietnamese social scientists diversify in terms of both publishers (see Figure 1a) and journals (see Figure 1b). The total number of unique publishers has grown substantially from 28 to 61 in 2019 since 2008, translating to a growth rate of up to 9.8% per annum while the number of sources had emerged rapidly by approximately 14.1% per annum. However, the growth rate of publishers and journals was fluctuating -14%–21%, and from 1% to 33% per annum, respectively. The rise in publishers and journals has both slowed down since 2017. In 2019, the increase was four publishers and three journals.

We also noticed a steady number of new publishers every year, which accounted for 25%–53% of the total number of publishers per year. However, since the total number of publishers kept increasing over the year, the percentage of new publishers has become more modest. The new journals also appeared in all years of the period 2008–2019; in 2016, in particular, the number of new journals peaked at 162. However, since 2017, the growth of new journals have decreased (only 150 new journals in 2019).

Table 3 shows the top ten journals by the number of articles by Vietnamese authors. Among these ten, five journals were about interdisciplinary research (*Sustainability, PLOS One, Journal of Development Studies, Asian Social Sciences, Culture, and Health & Sexuality*), three journals were about health and medicine sciences (International Journal of Environmental Research and Public Health, Global Health Action), and three journals were about Economics and Business (Management Science Letters, Applied Economics, and Journal of Risk and Financial Management). Subfields such as Health Economics and Public Health have both characteristics of social sciences and health sciences. Thus, they are included in the database as their contribution is significant.

**Table 3:** Top journals with the highest number of articles.

Source	Publisher	Publication	OA status^a^	Quartile 2019^b^
Sustainability	MDPI	56	OA	Q2
International Journal of Environmental Research and Public Health	MDPI	52	OA	Q2
PLOS One	PLOS	24	OA	Q1
Global Health Action	Taylor & Francis	23	OA	Q1
Management Science Letters	Growing Science	23	OA	Q2
Applied Economics	Taylor & Francis	21	Hybrid	Q2
Journal of Risk and Financial Management	MDPI	21	OA	N/A
Journal of Development Studies	Taylor & Francis	19	Hybrid	Q1
Asian Social Science	Canadian Center of Science and Education	18	OA	Q3/Q4
Culture, Health & Sexuality	Taylor & Francis	18	Hybrid	Q1

a Collected from the Directory of Open Access Journals and journals' homepage.

b Collected from The SCImago Journal & Country Rank: https://www.scimagoir.com/.

The sign of OA adoption in Vietnamese SSH is observable. Seven out of ten sources were OA journals, while the other three journals' publishing model was hybrid. In addition to that, one of the two publishers with the most journals on the list (MDPI) was a purely OA publisher. The top four journals, which were open access, had relatively high ranking compared to others on the list (Q1 and Q2).

#### 4.1.2 Open access

**Figure 2:**
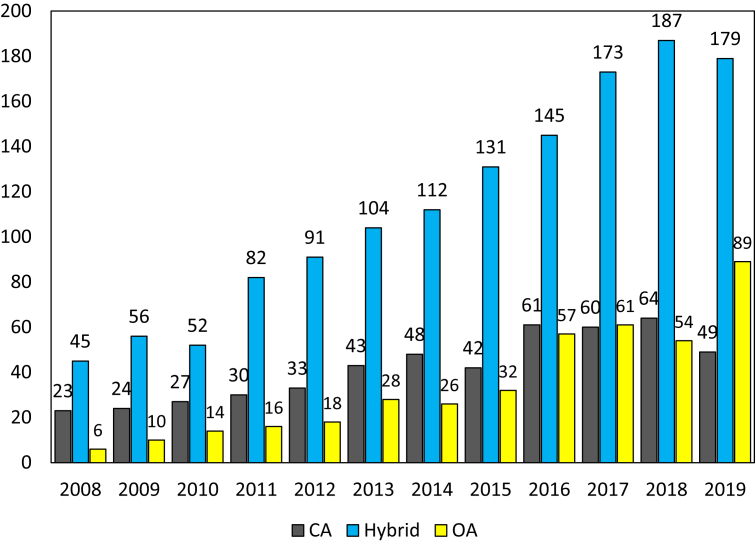
Number of sources by publishing models from 2008 to 2019.

Figure 2 illustrates the annual number of sources according to Open Access status (Open Access journal, Hybrid journal, and Closed Access journal). Among a total of 1371 different sources, except for 17 journals whose status was unknown, the number of Hybrid Journal is the highest (733 journals), nearly twice the Closed Access (392) and Open Access (246). In addition, the number of open access journals also rose swiftly from 6 journals in 2008 to 89 journals in 2019. Meanwhile, in the same period, the number of Closed Access journals only varied slightly, from 23 to 47. Notably, the number of Open Access sources was also equal to or higher than that of Closed Access sources in recent years (2016, 2017, and 2019). However, these findings are not concrete enough to conclude that Vietnamese SSH scientists are following the OA movement, so a finer view at the OA status at the article level might strengthen this presumption.

**Figure 3:**
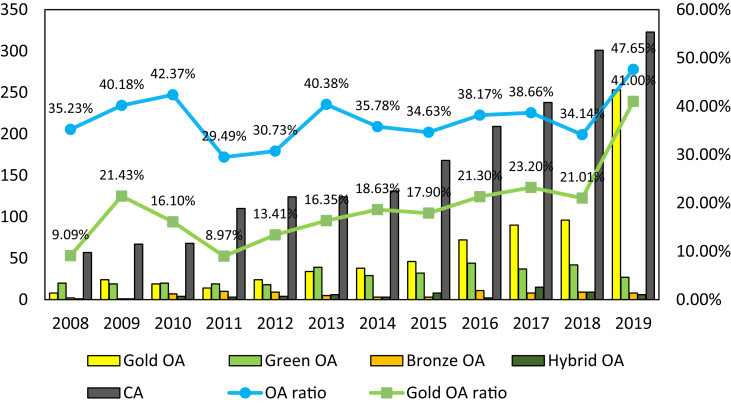
Number of articles by OA status during 2008–2019.

During the 2008–2019 period, the database recorded 3,122 publications that were published under five different types of Open Access: Gold, Green, Bronze, Hybrid, and Closed (See Figure 3). Although the percentage of OA publications increased slightly from 35.23% to 47.65% after 11 years, the increasing proportion of Gold OA publications from 9.09% to 41.00% was substantial. The selection of Gold OA rather than other modes of OA became very transparent in 2019, in which 253 over 294 OA publications were Gold standard. Given the superior meaning of Gold OA standards over other modes of OA, these findings hint that Vietnamese SSH scientists are progressively participating in the OA movement.

### 4.2 Bayesian analysis

#### 4.2.1 Open access model

**Figure 4:**
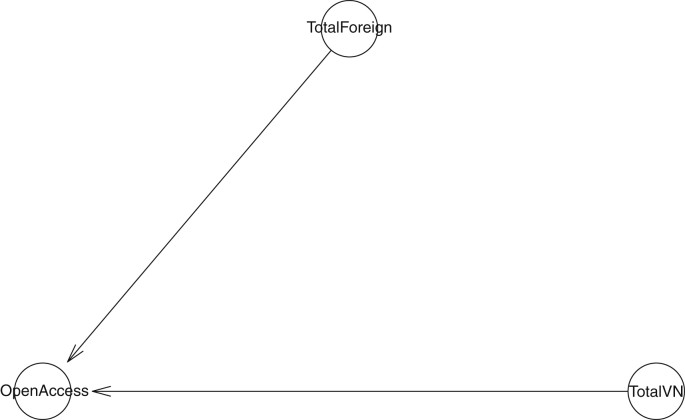
The Open Access journal model.

For examining the influence of the number of Vietnamese and international authors on the OA status of the publication, we construct the model as shown in Figure 4 and simulate the posterior distribution of all parameters in the model. The Stan code generated by the *bayesvl* package is available in the Supplementary.

The simulated results are shown in Table 4. All the effective sample sizes (n_eff) are larger than 4000, indicating a high number of effective samples, and thus, a good signal of correlation between dependent and independent variables. Also, the Gelman shrink factor (Rhat) of all parameters is 1, showing the convergence of Markov chains. MCMC trace plot of the model also confirms the Markov property of the coefficients' distribution (see Supplementary) (Vuong, 2020a; McElreath, 2020).

**Table 4:** Bayesian simulation results.

Parameters	Mean	Standard Deviation	n_eff	Rhat
a_OpenAccess	-1.17	0.06	4982	1
b_TotalVN_OpenAccess	0.22	0.02	5538	1
b_TotalForeign_OpenAccess	0.15	0.02	6795	1

Simulation settings: 4 chains, each with iteration = 5000; warmup = 2000; thin = 1; post-warmup draws per chain = 3000, total post-warmup draws = 12000.

**Figure 5:**
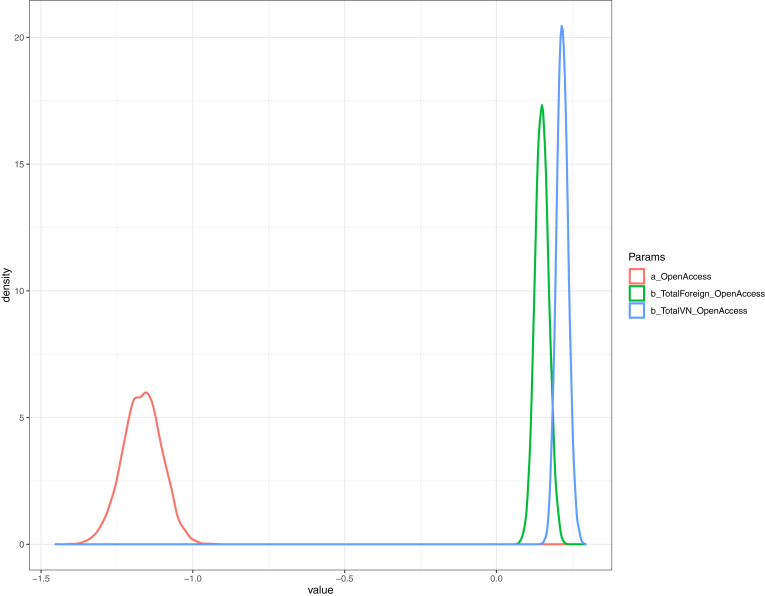
Coefficients' distribution of the model with OA decision as an outcome variable.

Both the number of Vietnamese and international authors (internal and external collaborative power, respectively) obtain a positive prediction of Open Access publishing outcome ($\beta_{TotalVN\_OpenAccess}$ = 0.22 and $\beta_{TotalForeign\_OpenAccess}$ = 0.15, respectively). This finding suggests that larger research teams or collaboration networks can improve the likelihood of adopting OA. Nevertheless, internal collaborative power is more influential than external collaborative power (see Figure 5).

## 5 Discussion

The current study's descriptive analysis employing data retrieved from the SSHPA database from 2008-2019 indicates several significant findings. First, Vietnamese social scientists have been diversifying journals and publishers for scientific publishing. Then, publishing in OA journals is one of the primary alternatives among Vietnamese social scientists. On the other hand, Bayesian simulation results highlight the significant impacts of internal and external collaborative power (measured by the number of domestic and international authors) and the likelihood of OA decision.

### 5.1 The diversification of publishing sources

Our finding indicated a surge in the number of sources and publishers in which Vietnamese social scientists published during 2008 and 2019. The surge can be explained by the top-down reform that requires not only natural sciences but also SS&H researchers to increase their scientific productivity and quality (Vuong, 2019b), which results in a higher demand for publishing in international journals among Vietnamese scholarly community. In particular, Circular 37/2014/TT-BKHCN issued by NAFOSTED in 2014 requires all principal investigators of national projects funded by NAFOSTED to result in ISI/Scopus publications. Moreover, the Circular 08/2017/TT-BGDDT issued by the government in 2017 revamps the requirements to complete the doctoral training program (Nguyen et al., 2019; Vietnam MOET, 2017). Subsequently, doctoral candidates have to obtain:

(i) at least two journal articles, one of them must be indexed by ISI Web of Science and/or Scopus; or
(ii) two reports at international peer-reviewed conferences; or
(iii) two articles in international peer-reviewed journals, for their dissertations, to qualify for the defense.

Previously, there was no requirement for international publications from Vietnamese authorities. Meanwhile, publications in domestic journals have been tainted by a personal relationship, plagiarism, or grey transactions (Vuong, 2018; Pham and Hayden, 2019). Consequently, the quality of scientific publications in domestic journals has been questioned by researchers who have been trained abroad. Raising the bar in requirements for national projects as well as doctoral qualification not only leads to the leveraged standard of doctoral candidates' mentors but also develops a ‘publish or perish’ mentality in the Vietnamese scholarly community. As a result, these policies have been empirically linked with an increase in SS&H research productivity (Nguyen et al., 2019; Vuong, 2019b). The detailed results have shown a continuous rise in the numbers of publishers and sources (See Figure 1). The rise suggests that Vietnamese SS&H researchers are finding new options that fit the requirements. However, the diversification of publishing sources also means a mixture of quality. In the top 10 journals with the highest number of articles, there is one Q3, one Q4, and one journal which has yet to be ranked. Indeed, lower quartiles do not necessarily mean lower quality. However, policymakers and university managers should pay attention to certain signs, such as a suspicious number of articles from a single country.

On the bright side, this policy is a necessary move to let young scholars experience and practice their skills in a peer-viewed process. Under certain circumstances, productivity is needed to raise quality because it increases the chance for good ideas to be created and executed (Sandström and van den Besselaar, 2016).

In addition to the policy reform, financial incentives granted by the government also played a major role in urging scientists to publish internationally. In 2008, the Vietnamese government initiated its first national scientific funding agency - the National Foundation for Science and Technology (NAFOSTED), which financially incentivizes and supports scientific projects. Six years later, the government issued Circular no. 23/2014/ND-CP to ensure that all scientific projects funded and sponsored by the Foundation complied with international standards (Tran et al., 2019). Given that many domestic journals are not of international standards (i.e., not indexed in either WoS, Scopus, or approved by NAFOSTED), these top-down approaches have broken the long-standing custom of social science research to publish in domestic journals and obliged scientists to diversify their publishing sources internationally. One way to export their research overseas is to collaborate with foreign colleagues. This approach also helps to increase their chance to publish in journals with higher JIF. This raises a concern over the capacity for self-reliance of Vietnamese authors when it comes to publishing scientific papers; in other words, the extent to which they are capable of publishing solo (Ho et al., 2020).

Although the trend to diversify journals is evident, not many Vietnamese authors challenge themselves with other types of publications than scientific articles. There are only 18 books, 141 edited books, and 20 conference proceedings published in a decade. Given the role of scholarly books in SS&H, policies encouraging researchers to write books is necessary, especially when book publications are suitable for SSH scholars whose explanations are usually long (Bonaccorsi, 2018; Engels et al., 2018).

### 5.2 Open-access publishing: a ‘short-cut’ to the global scientific standard?

The adoption of the OA publishing framework by the Vietnamese scientific community is a probable scenario. Effectively, we have found that seven out of ten journals that most frequently publish Vietnamese scientists' works during the period 2008–2019 were OA journals. Moreover, we also observe a rising proportion of gold OA articles in the examined period: from only 9.09% in 2008 to 41% in 2019. These impressive findings might result from the many benefits of publishing in an OA journal for scientists from emerging countries, like Vietnam. OA publishing is an opportunity for researchers from emerging countries to proliferate their scientific records due to the rapid processing time, higher recognition and rapid dissemination of research findings (Eysenbach, 2006; Harnad and Brody, 2004). For example, manuscripts are peer-reviewed and given a first decision approximately 17–17.6 days after submission into *Sustainability* and *International Journal of Environmental Research and Public Health*. Both journals are also indexed in many reputable databases, namely: Social Science Citation Index, MEDLINE, Scopus, EconPapers, IDEAS, and Chemical Abstracts, etc.

OA journals in which Vietnamese scientists publish are of quite high quality. Our study showed that among the top ten journals most frequently publishing Vietnamese research papers, four out of seven OA journals in the top ten are Q1 or Q2. The catch is that these journals often have an expensive article processing charge (APC) that is not affordable to many researchers in developing countries or ECRs. In particular, to publish an article in *Sustainability*, authors need to pay around $1,800 of APC, while the amount to publish in *PLOS One* is $1,595 of APC. Even though these journals and publishers often offer special programs to help authors from developing countries, the discount cost, which is around $500 per article, is still a considerable amount. Currently, no specific policy or regulation is enacted to financially support OA publishing, which currently hinders the OA movement in Vietnam by lifting the cost of doing science (Vuong, 2018).

Another concern of OA publishing apart from expensive APC is the quality of the journals. The pay-to-publish model of open access has created a loophole for predatory journals to exist and thrive. Many predatory journals disguise themselves as open-access journals and require authors to pay expensive APC to publish their paper, without editorial process or peer-review (Noorden, 2020; Grudniewicz et al., 2019). Currently, lists such as DOAJ (https://doaj.org/), Cabells' list (https://www2.cabells.com/about-predatory), or to a certain degree, Beall's list (https://beallslist.net/; discontinued) are valuable resources for researchers to cross-check the validity of a publisher or a journal. However, these lists have faced several criticisms, such as the case of Beall's list (Berger and Cirasella, 2015; Yeates, 2017), or are not publicly available (Cabells requires subscription).

Another striking finding is that internal collaborative power is more substantial in determining the decision to publish in OA journals than the external collaborative power. Based on this finding, we suspect a publishing pattern in which a group with a higher number of researchers are more likely to choose OA journals due to economic purposes. That pattern is substantially stronger if the additional authors are Vietnamese rather than foreign. The finding provides evidence for the assumption that Open Access publishing happens even more frequently to researchers from developing countries, like Vietnam, where researchers are usually evaluated depending mostly on the number, not quality, of publications (Bayry, 2013).

## 6 Conclusion and recommendations

The current study is the first study to use data from an exclusive database to examine the journals and publishers in which Vietnamese social scientists publish during the period 2008–2019. Our study showed the publication pattern of Vietnamese SSH researchers during the period with various changes in Vietnam science. We noticed that the Vietnamese scholarly community has gradually been accepting OA publishing, but there remain some challenges to achieve a sustainable scientific production system.

We recommend the government to implement, in due time, regulations and financial support for projects that integrate OA publishing framework, in order to not only promote the OA movement but also better assess the quality of OA journals. Currently, Plan S and its achievement in pushing open science are valuable lessons for Vietnam. A radical decision with collective supports from national and private funders is a right push toward the right direction. Thus, Vietnam's NAFOSTED should learn from the guidance and requirements of Plan S. On the other hand, ensuring scientific quality is also a critical mission. Paying for publications often denotes a negative meaning in the public's mindset. Therefore, the scientific community should be more open and engaging in communicating their results, ideas, and even failure to help the public understand (Vuong, 2020b).

Furthermore, the balance between internal and external collaborative power is necessary for promoting the OA movement and should be taken into consideration, especially for governments of emerging countries, when developing scientific policies. To facilitate the OA movement, a proactive attitude not only from the government but also from researchers is imperative (Vuong, 2019a).

## 7 Limitations

We fully acknowledge the shortcomings of this paper. Firstly, the paper used publication patterns to explore how Vietnamese SSH researchers are dealing with open access. The method only provides a single perspective. Thus, future studies might learn about the experiences of SSH researchers with OA publishing from a different perspective. Secondly, even though we attempted to discuss the question of quality, we were unable to do so because the metrics are still biased due to the different characteristics of different subject areas. For example, publications in Health Sciences might have higher JIF and number of authors per publications than other fields in Social Sciences, which might influence the result of the relationship between co-authorship and JIF. We hope to address this issue in the future. Finally, as our study only focuses on where Vietnamese social scientists published, we have neglected several aspects regarding the quality of OA journals and subject areas. Therefore, we recommend that future studies should pay more attention to these matters.

## Declarations

### Author contribution statement

T. T. Vuong: Performed the experiments; Wrote the paper.

H. Manh-Toan: Conceived and designed the experiments; Contributed reagents, materials, analysis tools or data; Analyzed and interpreted the data; Wrote the paper.

M. H. Nguyen: Conceived and designed the experiments; Performed the experiments; Wrote the paper; Analyzed and interpreted the data.

T. T. H. Nguyen, T. D. Nguyen, T. L. Nguyen, A. P. Luong: Contributed reagents, materials, analysis tools or data; Wrote the paper.

Q. H. Vuong: Conceived and designed the experiments; Performed the experiments; Analyzed and interpreted the data.

### Funding statement

This work was supported by 10.13039/100007224NAFOSTED - Vietnam National Foundation for Science and Technology Development (502.01–2018.19).

### Competing interest statement

The authors declare no conflict of interest.

### Additional information

No additional information is available for this paper.
