# Supplementary material for: Adopting open access in the social sciences and humanities: evidence from a developing nation
Source: Heliyon. 2020 Jul 23;6(7):e04522. doi: 10.1016/j.heliyon.2020.e04522 (PMC7387825; doi:10.1016/j.heliyon.2020.e04522)
Supplement: Supplementary [file mmc1.docx]

**Supplementary for: Adopting Open Access in the social sciences and humanities: Evidence from a developing nation**

**R Code:**

**1. Design the model**

| # Design the model  OA_m<-bayesvl()  OA_m<-bvl_addNode(OA_m,"OpenAccess","binom")  OA_m<-bvl_addNode(OA_m,"TotalVN","norm")  OA_m<-bvl_addNode(OA_m,"TotalForeign","norm")  OA_m<-bvl_addArc(OA_m,"TotalVN","OpenAccess","slope")  OA_m<-bvl_addArc(OA_m,"TotalForeign","OpenAccess","slope")  bvl_bnPlot(OA_m) |
| --- |

**2. Model check**

| > summary(OA_m)  Model Info:  nodes: 3  arcs: 2  scores: NA  formula: OpenAccess ~ a_OpenAccess + b_TotalVN_OpenAccess * TotalVN + b_TotalForeign_OpenAccess * TotalForeign |
| --- |

**3. Stan code**

| functions{  int numLevels(int[] m) {  int sorted[num_elements(m)];  int count = 1;  sorted = sort_asc(m);  for (i in 2:num_elements(sorted)) {  if (sorted[i] != sorted[i-1])  count = count + 1;  }  return(count);  }  }  data{  // Define variables in data  int<lower=1> Nobs; // Number of observations (an integer)  int<lower=0,upper=1> OpenAccess[Nobs]; // outcome variable  real TotalVN[Nobs];  real TotalForeign[Nobs];  }  transformed data{  // Define transformed data  }  parameters{  // Define parameters to estimate  real a_OpenAccess;  real b_TotalVN_OpenAccess;  real b_TotalForeign_OpenAccess;  }  transformed parameters{  // Transform parameters  real theta_OpenAccess[Nobs];  for (i in 1:Nobs) {  theta_OpenAccess[i] = a_OpenAccess + b_TotalVN_OpenAccess * TotalVN[i] + b_TotalForeign_OpenAccess * TotalForeign[i];  }  }  model{  // Priors  a_OpenAccess ~ normal(0,100);  b_TotalVN_OpenAccess ~ normal( 0, 10 );  b_TotalForeign_OpenAccess ~ normal( 0, 10 );  // Likelihoods  OpenAccess ~ binomial_logit(1, theta_OpenAccess);  }  generated quantities {  // simulate data from the posterior  int<lower=0,upper=1> yrep_OpenAccess[Nobs];  // log-likelihood posterior  vector[Nobs] log_lik_OpenAccess;  for (i in 1:num_elements(yrep_OpenAccess)) {  yrep_OpenAccess[i] = binomial_rng(OpenAccess[i], inv_logit(theta_OpenAccess[i]));  }  for (i in 1:Nobs) {  log_lik_OpenAccess[i] = bernoulli_logit_lpmf(OpenAccess[i] \| theta_OpenAccess[i]);  }  } |
| --- |

**4. Model simulation summary**

| > summary(OA_m)  Model Info:  nodes: 3  arcs: 2  scores: NA  formula: OpenAccess ~ a_OpenAccess + b_TotalVN_OpenAccess * TotalVN + b_TotalForeign_OpenAccess * TotalForeign  Estimates:  Inference for Stan model: 1a8d8b0dab5d59aecc69b2d41a1f8d21.  4 chains, each with iter=5000; warmup=2000; thin=1;  post-warmup draws per chain=3000, total post-warmup draws=12000.  mean se_mean sd 2.5% 25% 50% 75% 97.5% n_eff Rhat  a_OpenAccess -1.17 0 0.06 -1.30 -1.21 -1.17 -1.12 -1.04 4982 1  b_TotalVN_OpenAccess 0.22 0 0.02 0.18 0.20 0.22 0.23 0.26 5538 1  b_TotalForeign_OpenAccess 0.15 0 0.02 0.11 0.13 0.15 0.16 0.19 6795 1  Samples were drawn using NUTS(diag_e) at Tue Jun 30 12:46:54 2020.  For each parameter, n_eff is a crude measure of effective sample size,  and Rhat is the potential scale reduction factor on split chains (at  convergence, Rhat=1).  elapsed time: 310.198658943176 secs |
| --- |

**5. Visual diagnostics of MCMC chains of the Open Access model**

**
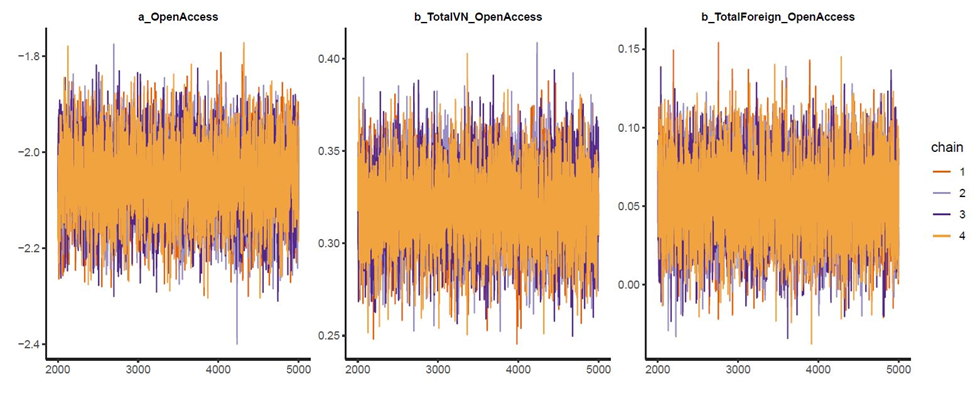
**
